# Investigation of the Immunological Profile of Children with Infectious Mononucleosis

**DOI:** 10.3390/children13101325

**Published:** 2026-09-29

**Authors:** Zhuo Sun, Zhihui Ning, Ning Han, Xiaoshan Li, Guangwan Lian, Yi Chen, Tao Lin, Mingqi Zhao, Bing Zhu

**Affiliations:** 1 Center Laboratory, Guangzhou Women and Children’s Medical Center, Guangzhou Medical University, Guangzhou 510120, China; 2 Department of Pediatrics, The Second Hospital, University of South China, Hengyang 421001, China

**Keywords:** infectious mononucleosis, Epstein–Barr virus, T lymphocyte, cytotoxic function, granzyme B, perforin

## Abstract

**Highlights:**

**What are the main findings?**

- Children with acute IM show marked CD8+ T- and NK cell expansion with a broad, Th1-skewed cytokine elevation (IL-12p70 unchanged).
- Granzyme B and perforin are strongly upregulated in CD8+ T and NK cells, indicating a pronounced cytotoxic-effector phenotype.

**What are the implications of the main findings?**

- These descriptive findings confirm and integrate the expected acute anti-EBV immune signature in a single pediatric cohort.
- They generate hypotheses—requiring prospective, controlled validation—about using this immune profile to support diagnosis and grade disease activity.

**Abstract:**

**Background/Objectives**: This study aimed to characterize the cell-mediated immune (CMI) profile—peripheral lymphocyte subsets, a 14-cytokine panel, and intracellular granzyme B/perforin expression—in children with acute IM compared with age- and sex-matched healthy controls, and to describe its dynamics at symptom resolution in an exploratory paired subset. This is a descriptive, hypothesis-generating study. **Methods**: We enrolled 40 patients and 50 healthy controls, analyzing peripheral blood T cell subsets, intracellular granzyme B and perforin expression, and serum cytokines. Cross-sectional comparisons were made between the IM group and the healthy control group at diagnosis; in addition, paired samples obtained at clinical symptom resolution were available for a subset of 7 patients and were analyzed as an exploratory longitudinal comparison. **Results**: Patients showed significantly elevated leukocytes, lymphocytes, and monocytes compared to healthy controls. Immunophenotyping revealed a higher total T cell count, with a lower CD4+ frequency but higher absolute count, and markedly increased CD8+ frequency and count. The B cell frequency was lower, while the NK cell frequency was lower but its absolute count was higher. A broad elevation in measured cytokines was observed, with significantly higher levels of IL-1β, IL-2, IL-4, IL-5, IL-6, IL-8, IL-10, IL-17A, IL-17F, IL-22, TNF-α, TNF-β, and IFN-γ. A cytotoxic phenotype was also more pronounced, with increased proportions of granzyme B- and perforin-expressing cells among CD3+ T cells, CD3+CD8+ T cells, and CD3-CD16/56+ NK cells. **Conclusions**: In conclusion, pediatric IM is characterized by profound changes in immune cell numbers and proportions, serum cytokine levels, and the expression of cytotoxic-effector molecules. These descriptive findings largely confirm the expected acute anti-EBV response and, in particular, reveal a pronounced coordinated CD8+/NK cytotoxic-effector signature. They warrant prospective, controlled study—ideally including a non-IM febrile/viral comparator—to determine whether such CMI parameters can support diagnosis or grade disease activity.

## 1. Introduction

Infectious mononucleosis (IM), primarily caused by Epstein–Barr virus (EBV), significantly impacts children and adolescents [1,2]. EBV exhibits a high global seropositivity rate, exceeding 90% in adults, and most children in developing regions are infected early in life [3,4]. Typical symptoms of EBV infection include fever, pharyngitis, hepatosplenomegaly, and lymphocytosis [3], but its expanding spectrum of atypical manifestations poses challenges for clinical diagnosis and management [5]. Severe complications, including associations with lymphoid and epithelial malignancies, underscore the need to elucidate its pathogenesis [6].

To date (as of 2026), no EBV vaccine has been licensed for children or adults, and the management of IM remains largely supportive; antivirals such as acyclovir and valacyclovir reduce oropharyngeal viral shedding but have limited effect on the clinical course. Several prophylactic and therapeutic candidates are, however, in clinical development. These include the mRNA vaccine mRNA-1189 (Moderna), evaluated in adolescents and young adults (12–30 years) and designed to target multiple viral surface glycoproteins to prevent primary infection and IM; the therapeutic mRNA candidate mRNA-1195 (Equinox) in adults, aimed at long-term complications such as post-transplant lymphoproliferative disorder and related malignancies; and an EBV gp350–ferritin nanoparticle vaccine assessed in young adults (18–29 years) to block viral entry (NCT04645147) [7]. Against this backdrop, a detailed CMI characterization of acute pediatric IM is relevant both for understanding protective effector responses and for informing immune endpoints of future vaccine and immunotherapy trials.

EBV infection is initiated through salivary transmission, infecting B lymphocytes and epithelial cells [8,9]. The virus triggers complex immune responses, reshaping lymphocyte populations [10,11,12]. During acute infection, EBV establishes latency and immortalization in B lymphocytes, while CD8+ T cells expand significantly, producing numerous atypical lymphocytes—a hallmark of IM [13]. Cytotoxic T lymphocytes (CTLs) and natural killer (NK) cells are critical effector cells in clearing infected cells by releasing perforin and granzyme B, with perforin forming pores in target cell membranes and granzyme B inducing apoptosis [13,14,15]. Additionally, Th1/Th2 immune imbalance is closely linked to disease prognosis [16]. These immune responses collectively form the host’s primary defense against EBV, explaining its persistent latency in B lymphocytes and lifelong carriage [17,18].

Despite advances in understanding the molecular mechanisms of EBV immune responses, the precise alterations in lymphocyte subsets, key cytokines, and intracellular granzyme B and perforin expression from symptom onset to resolution are unclear. Therefore, this study aimed to characterize the CMI profile—lymphocyte subsets, a 14-cytokine panel, and intracellular granzyme B/perforin expression—in children with acute IM compared with age- and sex-matched healthy controls, and to describe its dynamics at symptom resolution in an exploratory paired subset. We hypothesized that pediatric IM is characterized by a coordinated CD8+/NK cytotoxic-effector signature accompanied by a mixed, Th1-skewed cytokine response, and we discuss whether such parameters could, in adequately controlled future studies, help support the diagnosis of IM and grade disease activity. This is an exploratory, descriptive study; findings that confirm established biology are presented as such.

## 2. Methods

### 2.1. Study Design and Participants

This prospective observational study was conducted at the Guangzhou Medical University Affiliated Women and Children’s Medical Center between July and November 2023 and enrolled children with IM and healthy controls. This study was approved by the Ethics Committee of the Affiliated Women and Children’s Medical Center of Guangzhou Medical University (approval #2020-43001). Written informed consent was obtained from the legal guardians of all individual participants included in the study. All procedures were performed in accordance with the ethical standards laid down in the 1964 Declaration of Helsinki and its later amendments, as well as relevant national and institutional guidelines.

The diagnosis of IM requires integrating clinical manifestations with laboratory serological evidence [19]. This study defines IM diagnostic criteria as the presence of at least three clinical signs or symptoms alongside serological evidence of acute EBV infection. Clinical signs or symptoms include fever, pharyngitis, cervical lymphadenopathy, hepatomegaly, splenomegaly, or eyelid edema. Serological confirmation relies on positive Epstein–Barr virus capsid antigen IgM (EBV-VCA-IgM) antibodies, with positive EBV-VCA-IgG and negative EBV nuclear antigen IgG (EBV-NA-IgG) as supporting evidence. These criteria ensure that enrolled patients are in the acute phase of EBV infection.

Healthy children who underwent health check-ups at the Pediatric Health Clinic of the same center during the same period were enrolled as healthy controls. They had no recent symptoms of infection such as fever, sore throat, cough, runny nose, lymphadenopathy, or hepatosplenomegaly. Previous infections (more than 2 months ago) were fully resolved, and no antibiotics or antiviral medications were taken recently. Healthy controls were screened by both clinical history and laboratory testing, including a complete blood count; in addition, EBV-specific antibodies were tested in all healthy controls and were negative, indicating that the healthy control group was EBV-seronegative.

For all participants, the exclusion criteria were: (1) children presenting with organic diseases (e.g., cardiac, hepatic, or renal disorders), malignancies, genetic metabolic disorders, or congenital immunodeficiency conditions; (2) diagnosed with concurrent infectious disease; or (3) insufficient or incomplete clinical documentation.

Longitudinal (paired) sampling. All 40 patients were sampled at diagnosis, before antiviral treatment was initiated. Paired follow-up samples were obtained during the same hospital admission in 7 of the 40 patients; follow-up samples were not available for the remaining patients because of the limited inpatient window and constraints on repeat blood collection. The interval between the baseline and follow-up samples was approximately 3–7 days (from admission to discharge). The follow-up time point was defined by resolution of the presenting clinical features—primarily defervescence and improvement of pharyngitis, and, where present at baseline, regression of lymphadenopathy and reduction of hepatosplenomegaly—which determined the timing of the follow-up blood collection. A concurrent decrease in serum transaminases was observed as a supportive laboratory indicator but was not used to define the time point. All 7 patients received antiviral therapy with acyclovir or valacyclovir. Because the study did not include an untreated longitudinal comparator and IM is a self-limiting disease that improves over time, the paired changes are reported as changes “at follow-up” and are not attributed specifically to treatment. Whether these 7 patients are representative of the full cohort could not be established and is addressed as a limitation.

### 2.2. Clinical Data Collection and Blood Sample Collection

Clinical data, including demographic information and complete medical records, were systematically collected from the hospital’s electronic medical record system for all participants. Peripheral venous blood was drawn and processed according to experimental requirements. Blood samples for flow cytometry (including lymphocyte subset, granzyme B, and perforin detection) were collected directly in EDTA anticoagulant tubes. Separate EDTA tubes were used for plasma separation and quantitative EBV nucleic acid testing, and non-anticoagulant tubes were used for serum separation and cytokine and antibody detection. All plasma and serum samples were stored at −80 °C until analysis.

### 2.3. EBV DNA Quantification

DNA was extracted from plasma samples using a commercial EBV nucleic acid detection kit (Guangdong Huayin Pharmaceutical Technology Co., Ltd., Guangzhou, China). EBV DNA viral load was quantified by real-time quantitative PCR (qPCR) with the kit’s primers and probes. The cycling parameters were: 50 °C for 2 min, 94 °C for 2 min, then 40 cycles of 94 °C for 10 s and 60 °C for 35 s, with fluorescence monitored via the FAM/CY5 channels. Four EBV reference standards of varying concentrations were included in each run to establish a calibration curve. Viral loads are reported in IU/mL.

### 2.4. Anti-EBV Antibody Detection

EBV-specific antibodies (e.g., VCA-IgM) were detected by ELISA using a kit from EUROIMMUN Medizinische Labordiagnostika AG (Lübeck, Germany). Serum samples were diluted 1:100, added to pre-coated strips, and incubated for 30 min. Subsequently, an enzyme-labeled secondary antibody and substrate were added. After stopping the reaction, absorbance was measured at 450 nm to calculate the ISR (Immune Status Ratio) value. A positive sample was defined as ISR ≥ 1.1. This EUROIMMUN ELISA is a semi-quantitative assay reporting results as an Immune Status Ratio (ISR); kit-provided positive and negative calibrators/controls were included in each run and results were interpreted against the manufacturer’s validated cut-off (ISR ≥ 1.1). The assay was performed following the manufacturer’s Instructions for Use under the laboratory’s routine quality-control procedures.

### 2.5. Lymphocyte Subgroup Analysis

The proportion and absolute count of peripheral blood lymphocyte subsets were determined by flow cytometry using the BD MultiTEST IMK Kit with BD Trucount absolute-count tubes (BD Biosciences, San Jose, CA, USA) according to the manufacturer’s instructions. Briefly, EDTA-anticoagulated whole blood was stained in Trucount tubes with two antibody cocktails—CD3-FITC/CD4-APC/CD8-PE/CD45-PerCP and CD3-FITC/CD19-APC/CD16+56-PE/CD45-PerCP—followed by red-cell lysis. Samples were acquired on a B FACSCanto II and analyzed with FACSCanto software (version 8.0.3). The percentage and absolute count (cells/µL) of CD3+ T cells, CD3+CD4+ and CD3+CD8+ subsets, CD19+ B cells, and CD16+CD56+ NK cells were determined, with absolute counts derived directly from the counting beads in the Trucount tubes rather than from the complete blood count. Instrument performance was verified daily with BD FACSDiva CS&T beads, and reference intervals followed manufacturer and in-house values. In this study, T cell subsets were defined as CD3+CD4+ and CD3+CD8+; memory, naïve, and exhaustion phenotypes (e.g., CD45RA/CD62L, PD-1) were not characterized, which is noted as a limitation.

### 2.6. Cytokine Detection

Serum cytokines were measured with the AimPlex bead-based multiplex flow assay (Tianjin QuantoBio Bioscience Co., Ltd., Tianjin, China). A 14-cytokine panel was measured: IL-1β, IL-2, IL-4, IL-5, IL-6, IL-8, IL-10, IL-12p70, IL-17A, IL-17F, IL-22, TNF-α, TNF-β, and IFN-γ. Calibrators were diluted in a 1:3 gradient to eight concentrations to generate a standard curve, with kit standards and controls run in each batch and results reported in pg/mL. Following incubation of capture beads with the sample, PE-labeled detection antibodies were added. Fluorescence intensity was then analyzed using a flow cytometer to calculate cytokine concentrations.

### 2.7. Granzyme B and Perforin Detection

Intracellular granzyme B and perforin were assessed with a six-color kit (Qingdao Raisecare Biotechnology Co., Ltd., Qingdao, China) according to the manufacturer’s instructions. Fifty microliters of EDTA-anticoagulated whole blood was incubated with 25 µL surface antibody mixture (CD45-PE-Cy7, CD3-PerCP, CD8-APC-Cy7, CD16-APC, and CD56-APC) for 15 min at room temperature in the dark. Samples were incubated with 120 µL fixative for 15 min, followed by 2 mL permeabilization reagent at 37 °C for 15 min. After centrifugation at 250× *g* for 5 min, the supernatant was removed and 10 µL intracellular antibody mixture (granzyme B-PE and perforin-FITC) was added for 15 min. Two hundred microliters of 1× lysing reagent was then added. Acquisition and analysis used a BD FACSCanto II and FACSDiva software (version 8.0.3). Lymphocytes were selected by CD45/side scatter as described in the original analysis, followed by CD3+ T cell, CD3+CD8+ T cell, and CD3-CD16/56+ NK cell gates. Granzyme B-positive and perforin-positive fractions were calculated separately within each parent gate. CD16 and CD56 shared a fluorescence channel. Per-run negative control tubes were included, and granzyme B+/perforin+ thresholds were set within each parent gate after lymphocyte gating. These measurements assess intracellular expression, not secretion, antigen specificity, or target-cell killing; the fraction simultaneously positive for both molecules was not quantified. Because these assays were performed with commercial kits according to the manufacturers’ protocols, individual antibody clone and catalogue numbers, the number of events acquired, compensation settings, the use of fluorescence-minus-one/isotype/unstained controls, and doublet and viability exclusion were not separately recorded; these are acknowledged as limitations.

### 2.8. Statistical Analysis

Continuous variables were first tested for normality using the default method in SPSS (Shapiro–Wilk/Kolmogorov–Smirnov). Normally or approximately normally distributed data are presented as mean ± standard deviation and were compared using Student’s *t*-test (unpaired) or the paired *t*-test for the longitudinal analyses; variables that did not meet the normality assumption were compared using non-parametric tests (Mann–Whitney U test for unpaired comparisons and the Wilcoxon signed-rank test for paired comparisons). Categorical data are presented as *n* (%) and were analyzed using the chi-squared test or Fisher’s exact test. Because of the small sample size, the large number of variables compared without correction for multiple comparisons, and the exploratory nature of the paired analysis in only 7 patients, all analyses are exploratory and hypothesis-generating; the *p*-values should be interpreted accordingly and require confirmation in adequately powered studies. All analyses were performed using Prism 8.0 (GraphPad Software Inc., San Diego, CA, USA) and SPSS 27.0 (IBM, Armonk, NY, USA). Two-sided *p*-values < 0.05 were considered statistically significant.

## 3. Results

### 3.1. Characteristics of the Participants

Forty children diagnosed with IM were included in the IM group, comprising 27 boys and 13 girls, aged between 0.75 and 12 years (mean age, 4.97 ± 2.95 years). Fifty healthy children were included in the healthy control group, comprising 29 boys and 21 girls, aged between 1 and 8 years (mean age, 3.83 ± 2.09 years). There were no significant differences in age or sex between groups. The clinical manifestations and routine laboratory tests are presented in Table 1.

### 3.2. White Blood Cell Profile in IM

**Figure 1:**
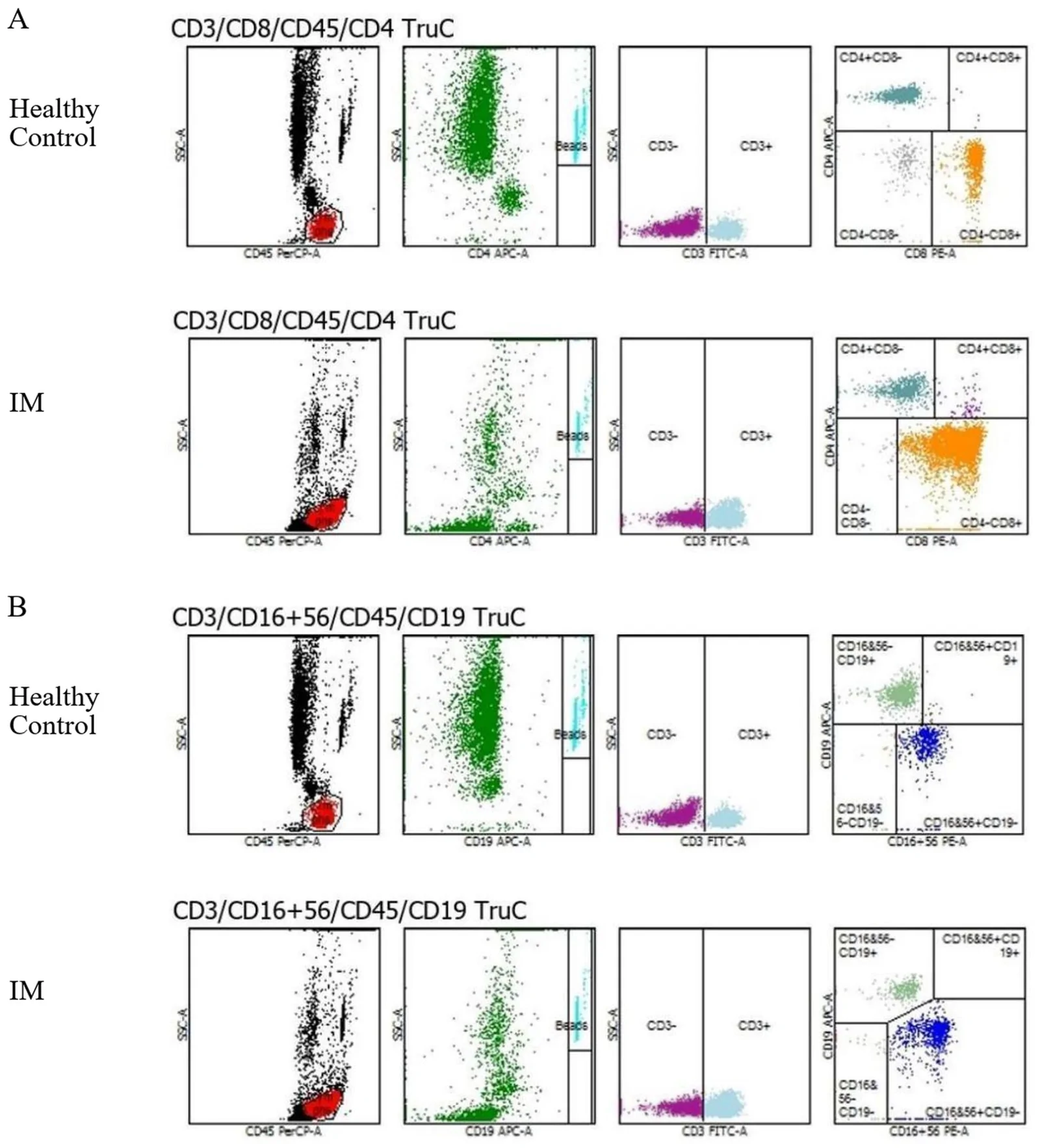
Flow cytometry graph of lymphocyte subpopulations. (**A**) CD3/CD8/CD45/CD4 panel. (**B**) CD3/CD16+CD56/CD45/CD19 panel. The upper and lower rows in each panel correspond to healthy controls and IM, respectively. CD45/side-scatter displays identify the lymphocyte region, followed by CD3 and subset-marker displays. These plots illustrate phenotypic identification rather than direct measurement of EBV infection or T cell proliferation. Table 2 provides group-level summary results (IM, *n* = 40; healthy controls, *n* = 50); the displayed plots are not pooled cohort data or evidence of individual age matching.

Compared with the healthy controls, the patients with IM showed higher leukocytes (14.28 ± 5.16 vs. 7.48 ± 1.28 × 10^9^/L, *p* < 0.001), lymphocytes (9.73 ± 3.96 vs. 4.04 ± 1.13 × 10^9^/L, *p* < 0.001), and monocytes (0.93 ± 0.56 vs. 0.43 ± 0.11 × 10^9^/L, *p* < 0.001). In addition, compared with healthy controls, children with IM showed higher T cell frequency and count, CD4+ cell count, CD8+ cell frequency and count, and NK cell count (all *p* < 0.001), while they showed lower CD4+ cell frequency, B cell frequency, and NK cell frequency (all *p* < 0.001) (Table 2 and Figure 1).

### 3.3. Cytokine Levels

**Figure 2:**
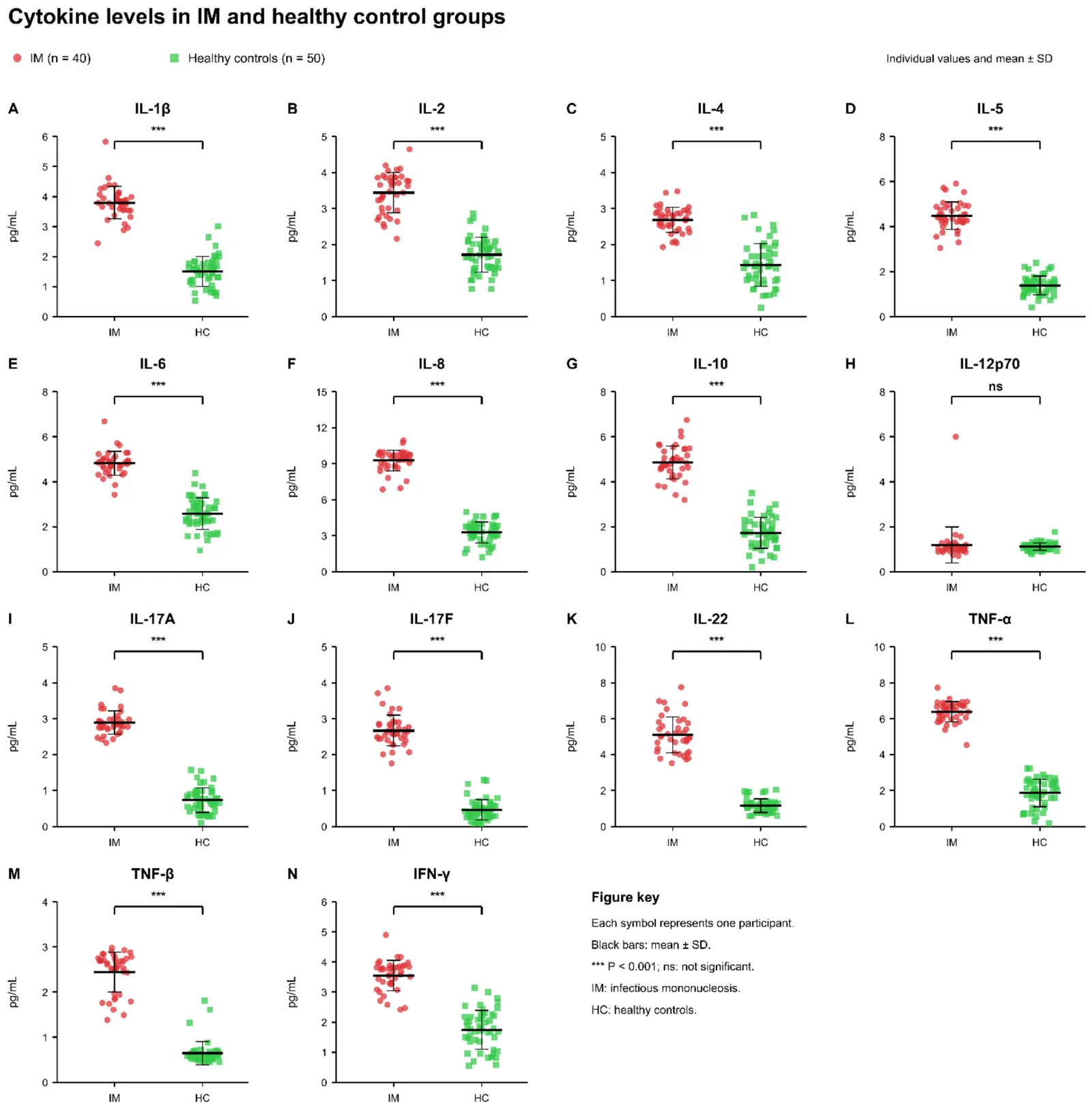
Cytokine levels in the infectious mononucleosis (IM) and healthy control groups. Levels of (**A**) IL-1β, (**B**) IL-2, (**C**) IL-4, (**D**) IL-5, (**E**) IL-6, (**F**) IL-8, (**G**) IL-10, (**H**) IL-12p70, (**I**) IL-17A, (**J**) IL-17F, (**K**) IL-22, (**L**) TNF-α, (**M**) TNF-β, and (**N**) IFN-γ in patients with infectious mononucleosis (IM; *n* = 40) and healthy controls (HC; *n* = 50). Red circles and green squares represent individual patients and healthy controls, respectively. Black horizontal bars indicate means, and error bars indicate standard deviations. Cytokine concentrations are expressed in pg/mL. *** *p* < 0.001; ns, not significant (IL-12p70, *p* = 0.095).

Compared with the healthy control group, the levels of IL-1β, IL-2, IL-4, IL-5, IL-6, IL-8, IL-10, IL-17A, IL-17F, IL-22, TNF-α, TNF-β, and IFN-γ were significantly higher in the IM group (all *p* < 0.001) (Figure 2). In contrast to the broadly elevated cytokines, IL-12p70 did not differ significantly between the IM and healthy control groups (*p* > 0.05).

### 3.4. Profiles of Granzyme B- and Perforin-Secreting Cells

**Figure 3:**
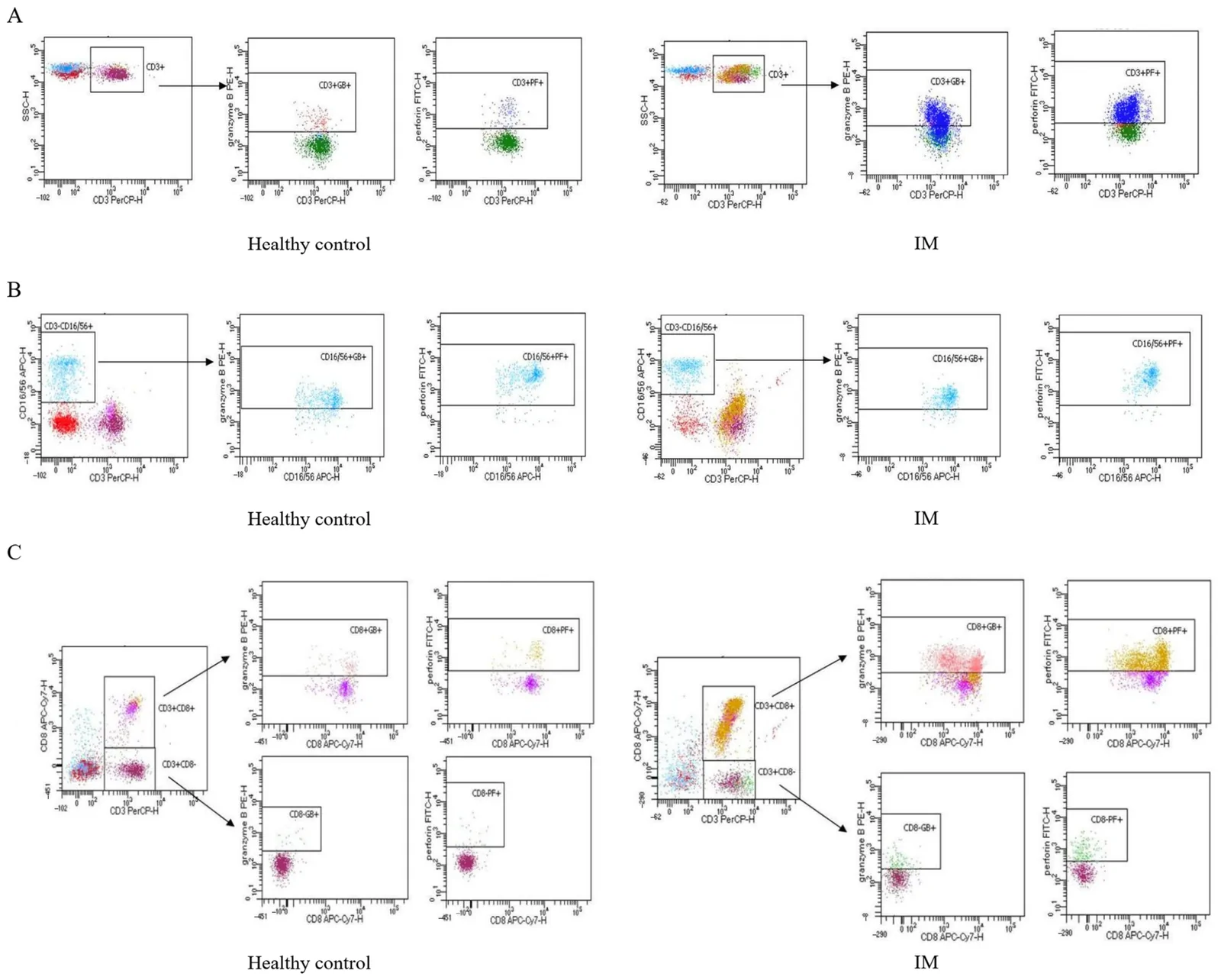
Flow cytometry gating strategy for granzyme B and perforin in the infectious mononcleosis (IM) and healthy control groups. (**A**) CD3+ T cells, (**B**) CD3-CD16/56+ NK cells, and (**C**) CD3+CD8+ T cells showed higher granzyme B and perforin expression in the IM group. Plots are representative; quantitative comparisons for all participants (IM group, *n* = 40; healthy control group, *n* = 50) are given in Figure 4, with between-group comparisons performed as described in Section 2.8.

**Figure 4:**
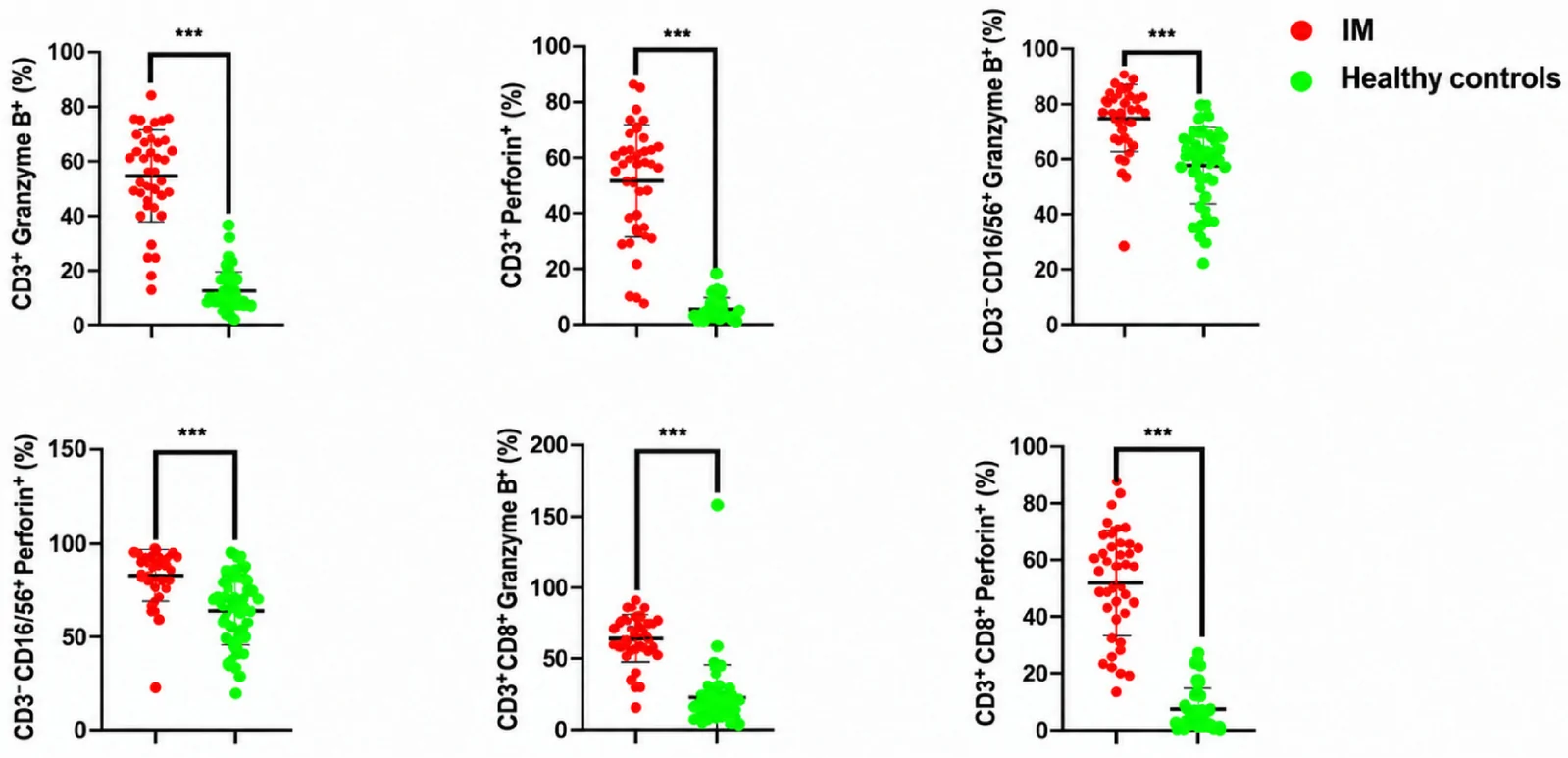
Frequencies of granzyme B- and perforin-secreting T Cells and NK cells between IM patients and healthy controls. Patients with infectious mononucleosis (IM; *n* = 40) were compared with healthy controls (*n* = 50). From left to right, the upper row shows the percentages of CD3+granzyme B+, CD3+perforin+, and CD3-CD16/56+granzyme B+ cells; the lower row shows the percentages of CD3-CD16/56+perforin+, CD3+CD8+granzyme B+, and CD3+CD8+perforin+ cells. Red and green symbols represent individual participants in the IM and healthy control groups, respectively. *** *p* < 0.001 for all comparisons.

Compared with the healthy control group, patients with IM showed higher proportions of granzyme B- and perforin-expressing cells among CD3+ T cells, CD3+CD8+ T cells, and CD3-CD16/56+ NK cells (i.e., CD3+ granzyme B+, CD3+ perforin+, CD3+CD8+ granzyme B+, CD3+CD8+ perforin+, CD3-CD16/56+ granzyme B+, and CD3-CD16/56+ perforin+; all *p* < 0.001) (Figure 3 and Figure 4). Because granzyme B and perforin were quantified as separate single-marker frequencies within each parent gate, the proportion of granzyme B+/perforin+ double-positive cells was not separately determined; this co-expression analysis is identified as a priority for future work.

### 3.5. Immune Cells and Cytokines at Follow-Up

In the exploratory paired analysis of 7 patients (baseline vs. follow-up at symptom resolution), the total T cell frequency and count and the CD8+ T cell frequency and count decreased significantly, while the CD4+ T cell frequency and the B cell frequency increased significantly (all *p* < 0.001). In contrast, the absolute CD4+ T cell count (*p* = 0.765), the absolute B cell count (*p* = 0.154), the NK cell frequency (*p* = 0.057), and the NK cell count (*p* = 0.052) did not change significantly (Supplementary Table S1). At follow-up, the serum levels of IL-1β, IL-2, IL-4, IL-5, IL-6, IL-8, IL-10, IL-12p70, IL-17A, IL-17F, IL-22, TNF-α, TNF-β, and IFN-γ decreased significantly (all *p* < 0.001) (Supplementary Table S2). Among the cytotoxic-molecule measurements, the proportions of CD3+ perforin+ (*p* = 0.009) and CD3+CD8+ perforin+ (*p* = 0.004) cells decreased significantly, whereas the proportions of CD3+ granzyme B+, CD3+CD8+ granzyme B+, and NK cell granzyme B+/perforin+ cells did not change significantly (all *p* > 0.05). EBV DNA copy levels decreased significantly (*p* = 0.017) (Supplementary Table S3).

## 4. Discussion

This study examined the immunological landscape of children with IM, showing that children with IM display significant elevations in lymphocyte counts, particularly CD8+ T cells and NK cells, which are pivotal in combating viral infections. The findings underscore the value of characterizing lymphocyte subsets and their granzyme B and perforin expression in understanding the immune response in IM, aiding in diagnosis and management. Many of our findings—leukocyte and lymphocyte expansion, a marked CD8+ predominance with an inverted CD4/CD8 ratio, and broad cytokine elevation—provide highly consistent confirmation of the expected acute anti-EBV response rather than a new mechanism, and we present them as such. Zhang et al. [20] characterized lymphocyte subsets and a limited set of cytokines in pediatric EBV-associated IM and reported CD8+ T cell expansion, an inverted CD4/CD8 ratio, and elevated inflammatory cytokines. Our results align with theirs on the cellular changes; the incremental contribution of the present study is the simultaneous, single-cohort assessment of lymphocyte subsets (with Trucount-based absolute counts), a 14-cytokine panel, and intracellular granzyme B/perforin across CD3+, CD3+CD8+, and NK compartments, together with an exploratory paired dataset linking cytotoxic-effector and viral-load dynamics at symptom resolution. The changes in immune cells, cytokines, and cytotoxic-molecule expression provide a more complete picture of the pathophysiology of IM and could ultimately suggest treatment targets.

This study investigated the alterations in the proportions and counts of the WBC types in children with IM, uncovering a notable elevation in lymphocyte counts, and that the surge in lymphocytes was mainly attributed to increased CD8+ T cells and NK cells. These alterations in lymphocyte subsets are consistent with the study by Zhang et al. [20]. CD8+ T cells, also known as CTLs, in conjunction with NK cells, play a crucial role in eradicating virus-infected cells during viral infections, thereby controlling the infection [21,22]. During IM, CD8+ T cells undergo profound alterations, reflecting a highly activated immune state and an intense effort to control EBV-infected B cells. There is a marked proliferation and expansion of CD8+ T cells, often resulting in an inverted CD4/CD8 ratio in peripheral blood [23,24]. These expanded populations can comprise a significant proportion of total lymphocytes and are a hallmark of the disease [24,25]. The presence of CD8+ T cells correlates with the severity of illness, high viral load, and abnormal liver function [25]. IM CD8+ T cells display high surface expression of inhibitory receptors associated with T cell activation and potential exhaustion, including PD-1, TIGIT, Tim-3, Lag-3, BTLA, 2B4, and KLRG1. Increased PD-1 and KLRG1 correlate with higher viral loads [25]. Despite phenotypic signs of activation and some markers of exhaustion, CD8+ T cells retain substantial proliferative and cytotoxic capacity during acute IM, helping to control viral replication. However, some studies observe impaired cytokine secretion capacity (IFN-γ, IL-2, TNF-α), suggesting a degree of functional exhaustion, likely due to persistent high antigen exposure [25]. There is an increased frequency of central memory phenotype (CD62L+ CD45RA-) and effector memory CD8+ T cells, while the frequency of naive CD8+ T cells decreases. Over time, after the resolution of acute infection, the proportion of memory CD8+ T cells increases, while effector populations decline [25,26]. There are often large monoclonal or oligoclonal expansions of CD8+ T cells, reflecting focused responses to specific EBV antigens [27,28]. Recent data suggest a gradual appearance of polyfunctional follicular CXCR5+CD8+ T cells as EBV viral load decreases, which may play a role in long-term control. In a similar manner to CD8+ T cells, NK cells during IM undergo selective expansion (most notably of early-differentiated, highly cytotoxic subsets) alongside heightened functional activation. These changes are central to controlling acute EBV infection, influencing disease severity, and modulating the broader immune response [29,30,31,32]. The lower B cell frequency observed in the IM group should be interpreted as a relative change: although the total lymphocyte count rose markedly, this was driven overwhelmingly by CD8+ T cell and NK cell expansion, so the proportion of B cells fell even though their absolute count did not differ significantly from that of healthy controls (Table 2). This pattern is consistent with EBV infection of B cells and the ensuing dominant cytotoxic T cell response directed against EBV-infected B lymphocytes. CD8+ T cells, NK cells, and especially CTLs mediate antiviral responses through the release of cytokines such as IFN-γ [33,34]. Monitoring the levels of IFN-γ and other cytokines is essential for evaluating the inflammatory status of children with IM.

This study showed that children with IM had elevated levels of IL-1β, IL-2, IL-4, IL-5, IL-6, IL-8, IL-10, IL-17A, IL-17F, IL-22, TNF-α, TNF-β, and IFN-γ compared with the healthy control group. These results are supported by Ye et al., who documented elevated IL-6 levels in EBV-infected children with IM [35], whereas Xu et al. reported increased levels of IL-17A and IL-22 in children with IM [36]. Similarly, Cai et al. identified increased IL-10 levels in children with IM [37]. IL-6 is significantly increased during the acute phase of IM in children compared to healthy controls [20,38]. TNF-α is markedly elevated during acute infection, exceeding levels in both healthy controls and individuals with other illnesses [38,39]. IL-10, an anti-inflammatory cytokine, is generally elevated during acute IM; however, its significance can vary between patients and studies [20,38,40]. IFN-γ is elevated in acute infection, reflecting strong Th1 immune activation [20,41]. IL-4 is also increased during the acute phase compared to convalescence, representing an enhanced Th2 response [20]. IL-18 is markedly elevated in the acute phase, particularly in the presence of severe inflammatory responses [42]. IL-27 is significantly higher in children with IM compared to healthy children, supporting its role in acute inflammation [43]. IL-1β, IL-2, and IL-8 may be measured but are often only sporadically elevated or less consistently reported in pediatric IM [38]. These findings suggest that dynamically tracking multiple cytokines may be informative for describing disease activity, although whether they can serve as validated indicators of disease progression or treatment response would require dedicated prospective studies. Overall, the cytokine pattern reflects a mixed but Th1-skewed response. The pronounced elevation of Th1-associated IFN-γ, IL-2, and TNF-α/β is consistent with antiviral CD8+/NK activation, and is accompanied by Th2-associated IL-4 and IL-5, Th17-associated IL-17A/IL-17F/IL-22, and the regulatory cytokine IL-10, indicating concurrent counter-regulation to limit immunopathology. This coordinated Th1/Th2/Th17 elevation, alongside a moderate ‘cytokine-storm’-like signature (IL-1β, IL-6, TNF-α, IFN-γ) balanced by IL-10, is characteristic of a vigorous yet self-limiting inflammatory response in immunocompetent children. Notably, IL-12p70 did not differ between groups. This was not unexpected: during acute EBV infection, IFN-γ is driven predominantly by antigen-experienced CD8+ T and NK cells and by IL-15/IL-18-dependent pathways rather than by systemic IL-12p70, so normal circulating IL-12p70 does not preclude strong Th1 activation, consistent with prior pediatric reports.

Beyond their antiviral activity mediated by the secretion of IFN-γ, CD8+ CTLs and NK cells primarily target and eliminate virus-infected cells through the release of granzymes and perforin [44,45]. This study revealed that IM children have elevated levels of granzyme B- and perforin-expressing CD8+ T cells and NK cells compared to their healthy counterparts, indicating an enhanced cytotoxic phenotype. This aligning with the observations reported by Zhang et al. [20]. The concurrent up-regulation of both granzyme B and perforin within the CD3+CD8+ and NK compartments indicates a fully armed cytotoxic program rather than isolated marker expression, since perforin-dependent membrane pore formation is the functional prerequisite for delivering granzyme B into EBV-infected B cells. Formal quantification of granzyme B+/perforin+ double-positive cells will be an important next step.

At follow-up (symptom resolution), EBV viral copy numbers decreased substantially, consistent with the literature [46]. Because this study did not include an untreated longitudinal comparator and IM is a self-limiting disease, these paired changes are reported as changes at follow-up and are not attributed to treatment. Alongside the falling viral load, the numbers of CD8+ T and NK cells declined, the proportion of perforin-expressing CD3+ and CD3+CD8+ cells decreased, and serum cytokine levels fell broadly [20,38,39]. A plausible interpretation is that, as viral antigen load subsides, the drive for cytotoxic-effector expansion and cytokine production wanes and effector-cell numbers contract. These observations suggest that dynamically monitoring lymphocyte subsets and cytokines could, if validated in adequately controlled studies, help track the clinical course of pediatric IM; given the very small paired subset (*n* = 7) and the absence of an untreated comparator, this remains exploratory and hypothesis-generating.

EBV alternates between latency and a lytic replication cycle. Lytic and immediate-early proteins (e.g., BZLF1) drive strong innate sensing and the release of IL-6, TNF-α, and IFN-γ, and expose immunodominant lytic antigens that shape the CD8+ response [47]; the intense cytotoxic and cytokine signature observed here therefore likely reflects, in part, active lytic replication during acute IM. When this normally self-limiting activation becomes dysregulated—through impaired immune control, host genetic susceptibility, or altered antigen presentation—chronic EBV-driven proliferation and immune stimulation can contribute to EBV-associated diseases. These include multiple sclerosis, for which large longitudinal data show a markedly increased risk after EBV seroconversion/IM [48] and mechanistic links via molecular mimicry between EBNA1 and CNS antigens [49], and Hodgkin lymphoma and other lymphoproliferative disorders [50]. The pronounced CD8+/NK cytotoxic profile we observed may thus be informative for understanding why most children clear EBV uneventfully while a minority go on to develop lasting immunological sequelae.

This study had limitations. The study was conducted at a single center with a small sample size, making the subgroup analyses susceptible to chance. The longitudinal (paired) analyses were based on only 7 of the 40 patients, sampled approximately 3–7 days apart during a single admission; these patients may not be representative of the full cohort, and the absence of an untreated comparator precludes attributing the observed changes to treatment. In addition, the wide age range of the children (0.75–12 years) was not stratified for age-specific immune response analysis, potentially overlooking developmental variations in immune function across different age groups. Additional limitations include the lack of a non-IM viral-infection (febrile) comparator, which limits inferences about disease specificity; incomplete treatment and follow-up data for the 33 patients without paired samples; the absence of exact symptom-onset-to-sampling intervals; the lack of EBV strain (type 1/2) typing; and no characterization of latent-versus-lytic infection or of memory subpopulations (TEM/TCM/TRM). Finally, this study did not correct for multiple comparisons, and the statistical significance of certain cytokine results may need further verification.

## 5. Conclusions

This descriptive study characterized the CMI profile of children with acute IM versus age- and sex-matched healthy controls, confirming a coordinated CD8+/NK cytotoxic-effector signature, a mixed Th1-skewed cytokine response, and elevated granzyme B/perforin expression. We propose the testable hypothesis that the intensity of this cytotoxic-effector signature tracks with disease activity and viral load. Rather than replacing current antiviral therapy, the practical value of such profiling—if validated prospectively against a non-IM viral comparator—would lie in supporting the diagnosis of IM, grading disease activity, and serving as an immune endpoint for future EBV vaccine and immunotherapy studies. A larger, multicenter, prospectively powered cohort with age stratification and latent/lytic and memory-subset characterization is warranted.

## Figures and Tables

**Table 1:** Baseline characteristics of the participants.

Index	Patient (*n* = 40)	Healthy Control (*n* = 50)	*p*
Age (mean ± SD), years	4.97 ± 2.95	3.83 ± 2.09	0.101
Age range, years	0.75–12	1–8	-
Sex			0.356
Male	27 (67.5%)	29 (58%)	
Female	13 (32.5)	21 (42%)	
Laboratory tests			
Leukocytes (×10^9^/L)	14.28 ± 5.16	7.48 ± 1.28	<0.001
Lymphocytes (×10^9^/L)	9.73 ± 3.96	4.04 ± 1.13	<0.001
Atypical lymphocytes %	13.45 ± 4.38	-	-
Neutrophils (×10^9^/L)	3.04 ± 1.51	2.74 ± 0.79	0.676
Monocytes (×10^9^/L)	0.93 ± 0.56	0.43 ± 0.11	<0.001
ALT (U/L)	80.88 ± 67.83	-	-
AST (U/L)	73.43 ± 52.01	-	-
Plasma EBV-DNA (IU/mL)	479.60 ± 258.28	-	-
VCA IgM	40 (100%)	-	-
VCA IgG	40 (100%)	-	-
EA IgG	37 (92.5%)	-	-
NA IgG	0 (0%)	-	-
Clinical manifestations			
Body temperature, °C	39.01 ± 0.67	-	-
Pharyngitis	40 (100%)	-	-
Lymph node enlargement	38 (95%)	-	-
Eyelid edema	30 (75%)	-	-
Liver enlargement	12 (30%)	-	-
Spleen enlargement	8 (20%)	-	-

ALT: alanine aminotransferase; AST: aspartate aminotransferase.

**Table 2:** Comparison of blood routine tests and lymphocyte subsets.

Blood Routine	Patients (*n* = 40)	Healthy Controls (*n* = 50)	*p*
Leukocytes (×10^9^/L)	14.28 ± 5.16	7.48 ± 1.28	<0.001
Lymphocytes (×10^9^/L)	9.73 ± 3.96	4.04 ± 1.13	<0.001
Atypical lymphocytes %	13.45 ± 4.38	-	-
Neutrophils (×10^9^/L)	3.04 ± 1.51	2.74 ± 0.79	0.676
Monocytes (×10^9^/L)	0.93 ± 0.56	0.43 ± 0.11	<0.001
T/Total lymphocytes %	83.48 ± 8.85	72.99 ± 5.83	<0.001
T cell count (cells/μL)	6383.95 ± 3083.59	1621.60 ± 467.84	<0.001
CD4+ T cells/total lymphocytes %	16.62 ± 8.13	37.98 ± 4.41	<0.001
CD4+ T cell count (cells/μL)	1164.75 ± 594.08	828.88 ± 247.99	<0.001
CD8+ T cells/total lymphocytes %	60.65 ± 16.11	28.40 ± 5.57	<0.001
CD8+ T cell count (cells/μL)	4750.06 ± 2466.53	636.62 ± 224.02	<0.001
B/Total lymphocytes %	7.71 ± 7.05	13.88 ± 3.22	<0.001
B cell count (cells/μL)	520.42 ± 507.07	340.50 ± 146.95	0.257
NK/Total lymphocytes %	8.23 ± 3.17	11.02 ± 3.84	<0.001
NK cell count (cells/μL)	656.89 ± 435.98	229.66 ± 99.05	<0.001

NK: natural killer.

## Data Availability

All data generated or analysed during this study are included in this published article.

## Supplementary Materials

The following supporting information can be downloaded at https://www.mdpi.com/article/10.3390/children13101325/s1, Table S1: Lymphocyte subset parameters in the IM group at baseline and follow-up; Table S2: Cytokine levels in the IM group at baseline and follow-up; Table S3: The dynamic changes in the secretion of granzyme B and perforin by T cells and NK cells, as well as the viral load, at baseline and follow-up.

## Abbreviations

Infectious mononucleosis (IM); Epstein–Barr virus (EBV); Cytotoxic T lymphocytes (CTLs); Natural killer (NK); Including interleukin (IL); Interferon (IFN); Mean fluorescence intensity (MFI).
