# Supplementary material for: Investigation of the Immunological Profile of Children with Infectious Mononucleosis
Source: Children (Basel). 2026 Sep 29;13(10):1325. doi: 10.3390/children13101325 (PMC13703288; doi:10.3390/children13101325)
Supplement: Supplementary file 1 [file children-13-01325-s001.zip › children-4547519-supplementary.pdf]

Supplementary Table S1. Lymphocyte subset parameters in the IM group at baseline and follow-up

Patients (n=7)	Baseline	Follow-up	P
T/Total lymphocytes %	87.79±2.84	79.54±2.56	<0.001
T cells count (cells/µL)	5785.79±1696.04	3649.40±946.19	<0.001
CD4+ T cells/Total lymphocytes %	12.58±2.82	25.99±3.43	<0.001
CD4+ T cells count (cells/µL)	832.25±279.78	836.86±225.13	0.765
CD8+ T cells /Total lymphocytes %	66.93±4.43	46.80±2.46	<0.001
CD8+ T cells count (cells/µL)	4460.70±1334.81	2515.22±680.01	<0.001
B/Total lymphocytes %	4.04±1.22	9.25±2.29	<0.001
B cells count (cells/µL)	252.30±69.26	306.16±131.67	0.154
NK/Total lymphocytes %	7.59±2.76	9.51±1.99	0.057
NK cells count (cells/µL)	502.07±270.09	351.07±132.61	0.052

Paired data from 7 IM patients. "Baseline" refers to sampling at diagnosis before antivirals; "follow-up" refers to sampling at symptom resolution (interval ≈ 3–7 days). All 7 received acyclovir or valacyclovir; with no untreated comparator and IM being self-limiting, changes are reported at follow-up and not attributed to treatment. Mean ± SD; paired t-test or Wilcoxon signed-rank test. No multiple-comparison correction; results are exploratory. NK: natural killer.

Supplementary Table S2. Cytokine levels in the IM group at baseline and follow-up

Patients (n=7)	Baseline	Follow-up	P
IL-1β (pg/mL)	3.79±0.30	1.96±0.18	<0.001
IL-2 (pg/mL)	3.41±0.43	1.93±0.26	<0.001
IL-4 (pg/mL)	2.84±0.43	1.58±0.18	<0.001
IL-5 (pg/mL)	4.49±0.57	2.23±0.26	<0.001
IL-6 (pg/mL)	4.87±0.58	2.96±0.66	<0.001
IL-8 (pg/mL)	9.21±0.51	4.86±0.59	<0.001
IL-10 (pg/mL)	4.53±0.56	2.40±0.33	<0.001
IL-12p70 (pg/mL)	1.01±0.08	0.85±0.06	<0.001
IL-17A (pg/mL)	2.81±0.27	1.40±0.19	<0.001
IL-17F (pg/mL)	2.68±0.55	1.26±0.22	<0.001
IL-22 (pg/mL)	5.38±0.94	2.54±0.35	<0.001
TNF-α (pg/mL)	6.48±0.34	3.24±0.40	<0.001
TNF-β (pg/mL)	2.52±0.35	1.25±0.13	<0.001
IFN-γ (pg/mL)	3.81±0.63	2.01±0.34	<0.001

Paired data from 7 IM patients. "Baseline" refers to sampling at diagnosis before antivirals; "follow-up" refers to sampling at symptom resolution (interval ≈ 3–7 days). All 7 received acyclovir or valacyclovir; changes are reported at follow-up and not attributed to treatment. Concentrations in pg/mL; mean ± SD; paired t-test or Wilcoxon signed-rank test. No multiple-comparison correction; results are exploratory. IL: interleukin; TNF: tumor necrosis factor; IFN: interferon.

Supplementary Table S3. The dynamic changes in the secretion of granzyme B and perforin by T cells and NK cells, as well as the viral load, at baseline and follow-up

Patients (n=7)	Baseline	Follow-up	P
CD3+ granzyme B+%	63.54±9.43	52.73±16.05	0.251
CD3+ perforin+%	61.06±16.33	31.60±13.58	0.009
CD3-CD16/56+ granzyme B+%	75.77±10.28	76.41±11.33	0.917
CD3-CD16/56+ perforin+%	84.77±9.98	78.43±13.35	0.372
CD3+ CD8+ granzyme B+%	69.74±8.13	64.70±13.83	0.504
CD3+ CD8+ perforin+%	60.31±22.15	26.00±15.83	0.004
EBV quantification (IU/mL)	519.57±438.46	160±152.69	0.017

Paired data from 7 IM patients. "Baseline" refers to sampling at diagnosis before antivirals; "follow-up" refers to sampling at symptom resolution (interval ≈ 3–7 days). All 7 received acyclovir or valacyclovir; changes are reported at follow-up and not attributed to treatment. Granzyme B+/perforin+ proportions (%) within each parent gate (single-marker; double-positive not determined); plasma EBV DNA by qPCR (IU/mL). Mean ± SD; paired t-test or Wilcoxon signed-rank test. No multiple-comparison correction; results are exploratory. EBV: Epstein-Barr virus.
